# 

**Published:** 1894-11-24

**Authors:** 


### CONTENTS.

**The Genesis and Treatment of Hyperpyrexia** - - - 127
**Unqualified Practice at Hospitals** - - - - - - 128
**The Future of Serum Therapeutics** - - - - - 129
**Studies in Applied Sociology.**—Emigrants: Whence they Come and Where they Go - - - - - - - - 130
**Annotations.**—The Dangers of Strong Tea—The Treatment of Intestinal Obstruction—The Trade in Remedies for Diphtheria - 131
**Notes and News** - - - - - - - - - - 132
**Scraps and Gleanings** - - - - - - - - - 142
**The Book World of Medicine and Science** - - - - 143
**Medical Progress and Hospital Clinics:**
Chronic Catarrh of the Middle Ear. VI.—Treatment (*concluded*) 133
Progress in Medicine.—Infective Diseases (*continued*) - - 135
Progress in Surgery.—Surgery of the Liver and Gall Bladder - 137
New Appliances and Things Medical - - - - - 138
**Editor's Letter-Box** - - - - - - - - 138
**The Institutional Workshop:**
Within the Hospitals.—The Radcliffe Infirmary, Oxford - 139
Practical Departments - - - - - - - - 141
Hospital Festivals and Meetings - - - - - - - 142

### EXTRA SUPPLEMENT.—THE NURSING MIRROR.

**News from the Nursing World.**—Our Princess in Russia—Lewisham Infirmary—Our Christmas Competition—Progress at Reading—Nursing as an Industry—The Blackburn Infirmary Needlework Guild—A Fresh Version—An Emergency Well Met—Queen's Nurses in Scotland—Wanted, a Little Help—Irish Entertainments—Short Items - - - xlviii
**The American Woman at Home.** II.—Her Food - - xlix
**Notes from Germany** - - - - - - - - - l
**Nursing in Military Hospitals** - - - - - - - l
**Dress and Uniforms** - - - - - - - - - lii
**Notes from St. Helena.** II.—First Visit to the Hospital - liii
**Hospitals in Natal** - - - - - - - - - - liii
**Appointments** - - - - - - - - - - - liii
**Wants and Workers** - - - - - - - - - liii
**In a Lying-in Hospital** - - - - - - - - liv
**Where to Go** - - - - - - - - - - - liv

